# Development and Evaluation of ABI-171, a New Fluoro-Catechin Derivative, for the Treatment of Idiopathic Pulmonary Fibrosis

**DOI:** 10.3390/ijms252111827

**Published:** 2024-11-04

**Authors:** Gian Luca Araldi, Yu-Wen Hwang, Ganesh Raghu

**Affiliations:** 1 Avanti Biosciences, Inc., 3210 Merryfield Row, San Diego, CA 92121, USA; 2 Department of Molecular Biology, New York State Institute for Basic Research in Developmental Disabilities, 1050 Forest Hill Road, Staten Island, NY 10314, USA; 3 Center for Interstitial Lung Diseases, University of Washington Medical Center, Seattle, WA 98195, USA

**Keywords:** fluoro-catechin derivatives, anti-pulmonary fibrosis, IPF treatment, DYRK1A, PIM1

## Abstract

The persistent challenge of idiopathic pulmonary fibrosis (IPF), characterized by disease progression and high mortality, underscores the urgent need for innovative therapeutic strategies. We have developed a novel small molecule—catechin derivative ABI-171—selectively targeting dual-specificity tyrosine-phosphorylation-regulated kinase 1A (DYRK1A) and proviral integration site for Moloney murine leukemia virus 1 (PIM1) kinases, crucial in the pathogenesis of fibrotic processes. We employed the Bleomycin-induced (intratracheal) mouse model of pulmonary fibrosis (PF) to evaluate the therapeutic efficacy of ABI-171. Mice with induced PF were treated QD with ABI-171, either prophylactically or therapeutically, using oral and intranasal routes. Pirfenidone (100 mg/kg, TID) and Epigallocatechin gallate (EGCG, 100 mg/kg, QD), a natural catechin currently in a Phase 1 clinical trial, were used as reference compounds. ABI-171, administered prophylactically, led to a significant reduction in hydroxyproline levels and fibrotic tissue formation compared to the control group. Treatment with ABI-171 improved body weight, indicating mitigation of disease-related weight loss. Additionally, ABI-171 demonstrated anti-inflammatory activity, reducing lymphocyte and neutrophil infiltration. In the therapeutic setting, ABI-171, administered 7 days post-induction, reduced mortality rates (*p* = 0.04) compared with the bleomycin and EGCG control groups. ABI-171 also ameliorated the severity of lung injuries assessed by improved Masson’s trichrome scores when administered both orally and intranasally. ABI-171 significantly decreases bleomycin-induced PF and improves survival in mice, showcasing promising therapeutic potential beyond current medications like pirfenidone and EGCG for patients with IPF. Based on these results, further studies with ABI-171 are ongoing in preclinical studies.

## 1. Introduction

Idiopathic pulmonary fibrosis (IPF) is a severe, progressive interstitial lung disease characterized by the abnormal and excessive deposition of fibrotic tissue within the lungs [1,2]. This fibrotic tissue disrupts the normal lung architecture, leading to increased stiffness and reduced elasticity, which impairs the lungs’ ability to facilitate efficient gas exchange. Clinically, IPF manifests as chronic, worsening dyspnea (shortness of breath), a persistent dry cough, and fatigue, significantly diminishing the quality of life and functional capacity of affected individuals. The prognosis for IPF is poor, with a median survival time of 3 to 5 years from diagnosis [1,2].

The pathogenesis of IPF is complex and not fully understood. It is believed to involve a combination of genetic predisposition and environmental exposures that leads to repeated injury and aberrant repair of alveolar epithelial cells (AECs). This results in the activation and proliferation of myofibroblasts, which produce and deposit extracellular matrix (ECM) proteins such as collagen, contributing to the progressive scarring (fibrosis) of lung tissue [3]. Transforming Growth Factor-beta (TGF-β), a key mediator of fibrosis, is released from injured AECs and other cells, promoting ECM production, deposition, and inhibiting ECM-degrading enzymes [4]. Additional signaling molecules, such as connective tissue growth factor (CTGF), platelet-derived growth factor (PDGF), and fibroblast growth factor (FGF), also contribute to the fibrotic process [5].

Currently, the treatment options for IPF are limited and primarily focused on slowing disease progression and managing symptoms. The two FDA-approved drugs for IPF, pirfenidone [6] and nintedanib [7], have been shown to slow the decline in lung function modestly. However, these treatments have significant limitations, including side effects that can impact patient adherence and their inability to improve overall survival or quality of life significantly [2]. There remains an urgent need for more effective therapies that can halt or reverse the fibrotic process and provide meaningful clinical benefits for patients with IPF.

Epigallocatechin gallate (EGCG), a natural catechin derived from green tea, has emerged as a potential therapeutic agent for IPF due to its anti-fibrotic and anti-inflammatory properties [8]. EGCG has been shown to inhibit several key pathways involved in fibrosis, including the TGF-β signaling pathway, which is critical for activating fibroblasts and producing ECM proteins [9]. By blocking TGF-β1 responses and reducing the expression of pro-fibrotic markers such as Snail and collagen, EGCG can mitigate the fibrotic process [10]. Additionally, EGCG has demonstrated the ability to reduce oxidative stress and inflammation, which are significant contributors to the pathogenesis of IPF [11].

Preclinical studies have shown that EGCG can reduce fibrosis in animal models of IPF, leading to improved lung function and reduced fibrotic markers. In a small human trial with IPF patients, EGCG was found to be effective in lessening fibrotic indicators [12]. Despite its promising anti-fibrotic effects, EGCG faces several challenges that limit its therapeutic potential. One of the primary limitations is its poor bioavailability, which restricts the amount of compound that can reach the target tissues in the lungs [13]. Furthermore, EGCG’s selectivity profile and safety concerns, particularly related to liver toxicity [14], pose additional hurdles to its development as a therapeutic agent for IPF.

To address these limitations, we have explored the development of novel derivatives of EGCG that retain their beneficial properties while improving bioavailability and safety [15,16]. ABI-171 (see Table 1), a novel fluoro-catechin derivative, has emerged as a promising candidate for the treatment of IPF. ABI-171 is a potent dual inhibitor of dual-specificity tyrosine-phosphorylation-regulated kinase 1A (DYRK1A) and proviral integration site for Moloney murine leukemia virus 1 (PIM1) kinases (this study). Both DYRK1A and PIM1 are serine/threonine kinases implicated in various cellular processes, including cell proliferation, survival, and differentiation [17,18].

The mechanism of action of ABI-171 potentially involves several key pathways and processes that contribute to its potential efficacy in treating IPF. DYRK1A plays a significant role in regulating neuronal development, cell cycle regulation, and stress response [17], while also being involved in the regulation of inflammation and fibroblast activation in IPF [19]. ABI-171’s inhibition of DYRK1A leads to the attenuation of inflammatory responses and a reduction in fibroblast activation—critical steps in the development of pulmonary fibrosis. By targeting DYRK1A, ABI-171 helps modulate the inflammatory environment within the lungs, thereby reducing the recruitment and activation of fibroblasts and myofibroblasts, the primary cells responsible for producing and depositing ECM proteins.

PIM1 kinase is another important target of ABI-171. PIM1 is involved in controlling cell survival, proliferation, and differentiation [18]. In IPF, PIM1 is upregulated and contributes to epithelial-to-mesenchymal transition (EMT), a process where epithelial cells lose their characteristics and gain mesenchymal, fibrogenic properties [20,21]. This is a crucial event in IPF, as it leads to the generation of myofibroblasts responsible for excessive collagen deposition. By inhibiting PIM1, ABI-171 may disrupt this process, thereby reducing the generation of myofibroblasts and the subsequent fibrosis. Inhibition of PIM1 has also been shown to alleviate lung fibrosis in a bleomycin-induced mouse model. Additionally, PIM1 inhibition decreases phosphorylation and activation of the nuclear factor of activated T cells (NFATc1) [22], a transcription factor promoting cell proliferation and survival, further contributing to ABI-171’s anti-fibrotic effects by reducing fibroblast proliferation and survival.

ABI-171, as a trihydroxyphenolic compound, could serve as a potential inhibitor of lysyl oxidase-like 2 (LOXL2) and, indirectly, impede TGF-β signaling [9]. Polyphenolic compounds inhibit LOXL2 activity by inducing auto-oxidation of specific lysine residues in LOXL2, preventing the enzyme from facilitating the conversion of lysine residues in collagen to aldehydes, reducing collagen cross-linking and tissue stiffening, both hallmarks of fibrosis [9].

Polyphenolic compounds also inhibit TGF-β signaling; however, the inhibition requires the presence of LOXL2. Apparently, inhibition of TGF-β signaling is mediated through the putative amino-catechol metabolites of polyphenolic compounds resulting from the interaction with LOXL2 [9]. Both LOXL2 and TGF-β signaling contribute to the elevation of Snail1, a transcription factor key to EMT. By inhibiting LOXL2 and TGF-β signaling, ABI-171 could reduce Snail1 expression and prevent EMT progression, thus mitigating fibrotic tissue development and the pathological collagen deposition seen in IPF.

This dual inhibition of LOXL2 and TGF-β signaling, combined with its inhibition of DYRK1A and PIM1 kinases, makes ABI-171 a promising candidate for therapeutic intervention in fibrotic diseases. Based on these mechanisms, we hypothesize that ABI-171 will significantly reduce fibrosis and improve lung function in preclinical models of IPF, providing superior efficacy compared to current treatments. This multifaceted mechanism of action highlights ABI-171’s potential as a disease-modifying agent for IPF, offering a promising therapeutic approach for this devastating disease.

This study aims to comprehensively evaluate ABI-171’s therapeutic potential for IPF. The objectives include characterizing its pharmacokinetic properties, assessing its therapeutic potential in preventive and therapeutic animal models, and elucidating its effects on key molecular pathways involved in IPF pathogenesis. The findings from this research will support the development of ABI-171 as a novel therapeutic agent for IPF and provide a foundation for subsequent clinical trials.

## 2. Results

In the study, two compounds, ABI-154 and ABI-171, were initially evaluated for their therapeutic potential in treating IPF (Table 1). ABI-154, a potent inhibitor of DYRK1A, exhibited strong efficacy in initial preclinical tests due to its ability to modulate key pathways involved in inflammation and fibrosis. Despite its high potency, ABI-154 faced challenges related to pharmacokinetics and bioavailability [16] (and Table 2), which limited its overall therapeutic impact. ABI-171, a fluoro-methyl catechin derivative, was further developed to address these limitations. The development was outlined in [16] and was based on the known observations that catechin methylation, such as in natural EGCG-3”OMe, results in much-improved bioavailability [23]. The modification enhances pharmacokinetics profiles [16] (and Table 2). In addition, ABI-171 not only targets DYRK1A but also inhibits PIM1 kinase, broadening its anti-fibrotic activity by disrupting multiple pathways involved in fibrosis progression, including EMT and fibroblast activation. This dual inhibitory action, coupled with improved pharmacokinetic properties, such as better bioavailability and a favorable safety profile, positions ABI-171 as a more promising candidate for IPF treatment. The comparative evaluation of these compounds in this study aims to highlight the efficacy of ABI-171 in supporting its potential as a disease-modifying therapy for IPF.

### 2.1. ABI-171 Pharmacokinetics and Lung-Tissue Distribution

The pharmacokinetics (PK) of ABI-171 and ABI-154 were evaluated in male C57BL/6 mice following single doses administered via intranasal (IN 60 mg/Kg), oral (PO 100 mg/Kg), and intravenous (IV 5 mg/Kg) routes. Plasma concentrations were measured using LC-MS/MS, and PK parameters were calculated with WinNonlin. To facilitate drug delivery, we have developed a proprietary liquid formulation that allows the delivery of these drugs in high concentrations for oral or intranasal use [15,16]. The formulation is based on the solubilizer and penetration enhancer (2-hydroxypropyl)-β-cyclodextrin (HP-β-CD), PEG-400, and water. This vehicle allows us to achieve high drug concentrations in the dosing solution (up to 20% *w*/*w*) when needed. PK studies for these compounds in plasma and the brain have been reported in an earlier publication [16]. Here we report the concentration of the drug in the lungs. As previously shown, despite the total drug exposure of ABI-171 in plasma being higher when the drug was delivered via IN than via PO (Table 2), the IN route is less efficient than the PO route in delivering a drug into tissues, such as into the brain [16]. In the lungs, however, the outcome is opposite, with the IN route shown to be highly superior to the PO route in delivering ABI-171 to the target (Table 2). Using the IN route, we can achieve a high and stable exposure in the lungs with a theoretical cellular concentration (~17.8 μM at C_max_), which far surpasses the IC_50_ for inhibiting DYRK1A and PIM1. Considering the C_max_ and the T_1/2_, it is possible that a single IN dose may still sustain a concentration of ABI-171 in the lungs above the IC_50_ for inhibiting DYRK1A and PIM1 after 24 h. However, it should be noted that the estimate is for the total, not the unbound ABI-171. To further confirm the PK results, we selected both ABI-154 and ABI-171 using the IN routes for the BLM efficacy studies.

### 2.2. Efficacy in Animal Models

#### 2.2.1. Preventative IPF Animal Model (Treatment Start at Day 0 After BLM Instillation)

**Figure 1:**
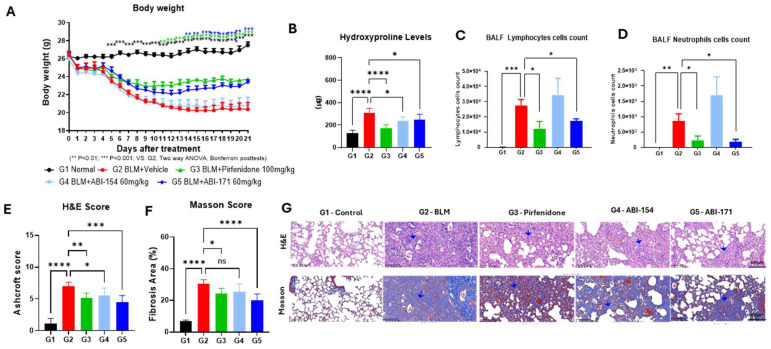
Preventative treatment study. (**A**) body weight; (** *p* < 0.01; *** *p* < 0.001; vs. G2, two-way ANOVA, Bonferroni post-tests); (**B**) hydroxyproline level; (**C**,**D**) lymphocytes and neutrophile assay; (**E**) H&E score; (**F**) Masson score; (**G**) representative histology pictures with a blue arrow indicating fibrosis with lung structural destruction. (**B**–**F**) Statistical analysis: ns (not significant), * *p* < 0.05; ** *p* < 0.01; *** *p* < 0.001, **** *p* < 0.0001 vs. G2 one-way ANOVA. (**B**–**F**) Data are presented as the mean ± standard error of the mean (*n* = 6).

PK studies using the IN route showed significantly elevated lung drug concentrations. Consequently, we opted for intranasal delivery in our first IPF animal model, employing a validated BLM mouse model. In this study, 30 male C57BL/6 mice were randomly assigned to five groups: Control (Normal), BLM (vehicle), pirfenidone (100 mg/kg, TD, oral), ABI-154 (60 mg/kg, QD, intranasal), and ABI-171 (60 mg/kg, QD, intranasal). The experiment considered the day of bleomycin injection as Day 0. The IPF model was successfully induced through a single intratracheal infusion of bleomycin. The experiment was completed after 21 days. Body weight loss induced by bleomycin was observed in the BLM group (*** *p* < 0.001), while the ABI-171 and the pirfenidone groups showed a significant increase in body weight from Day 14 to 21 (** *p* < 0.01; *** *p* < 0.001) and an improved delta body weight (* *p* < 0.05) compared to the model group (Figure 1A). On the other hand, ABI-154 had no body weight gain effect.

The collagen content in lung tissues was assessed by measuring the hydroxyproline content. Compared with the BLM group, the hydroxyproline content significantly decreased in the BLM + ABI-154 and ABI-171 groups (Figure 1B). Lymphocytes and neutrophils increased significantly in the BLM group but were significantly reduced in the ABI-171 group (*p* < 0.05), indicating a potent anti-inflammatory effect (Figure 1C,D). To explore the impact of our fluoro-catechins on bleomycin-mediated PF, pathological alterations in lung tissue were observed by H&E and Masson staining in each group. HE staining was used to determine fibrosis, while collagen deposition within different sections was analyzed by Masson’s trichrome staining (Figure 1E,F). After bleomycin administration, a remarkable increase in alveolar septa thickening, as well as collagen deposition within lung tissue, was observed (BLM group, Figure 1G). However, drug treatment had a reverse effect, with a decrease in the alveolitis and PF degrees relative to the bleomycin group (154 and 171 cohorts, Figure 1G). ABI-171 exhibited superior and positive effects on body weight, inflammation, hydroxyproline levels, and lung pathology in the context of IPF when compared to ABI-154. As a result, ABI-171 was selected for further investigation in follow-up studies (Section 2.2.2).

#### 2.2.2. Therapeutic IPF Animal Model (Treatment Start at Day 7 After BLM Instillation)

Based on the positive outcomes of our preliminary study, we designed a subsequent study to evaluate the therapeutic potential of ABI-171. In this study, drug treatment commenced 7 days post-induction of pulmonary fibrosis using BLM, employing a therapeutic model. We assessed the efficacy of ABI-171 administered intranasally at two dosages (10 mg/kg and 50 mg/kg, QD) and orally (100 mg/kg, QD), comparing it to oral EGCG (100 mg/kg, once daily), natural catechin currently undergoing Phase 1 clinical trials for IPF [24]. Unexpectedly, EGCG demonstrated limited to moderate activity in various assessments, notably a survival rate with 50% mortality. Conversely, both intranasal (50 mg/kg) and oral administration of ABI-171 resulted in complete survival and good recovery in body weight.

**Figure 2:**
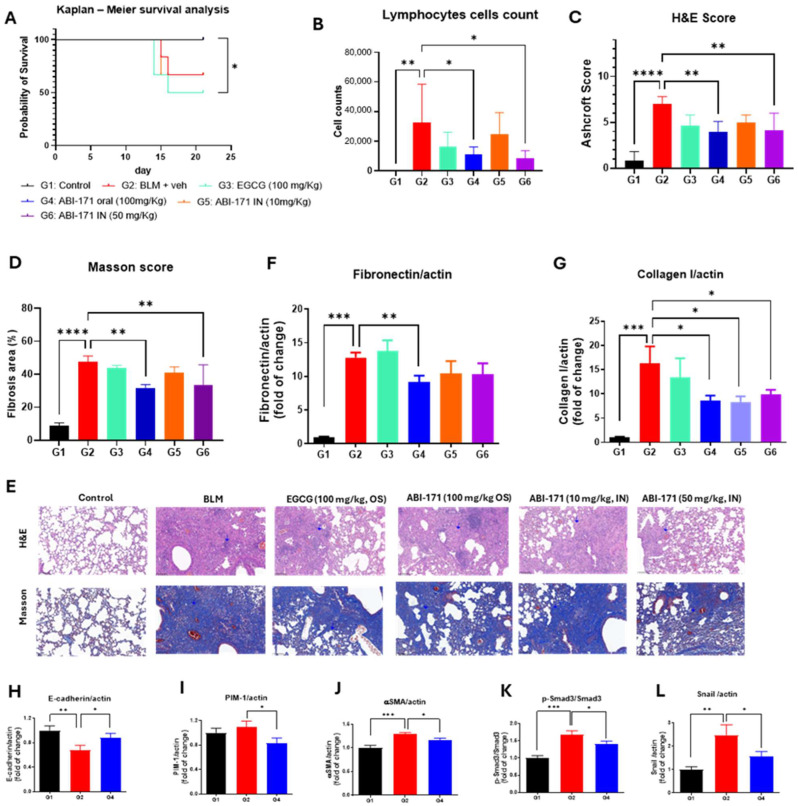
Therapeutic treatment study. (**A**) Kaplan–Meier survival curve, * *p* < 0.05; (**B**) lymphocyte cells count; (**C**) H&E score; (**D**) Masson score; (**E**) representative histology pictures with blue arrow indicating fibrosis with lung structural destruction; (**H**) fibronectin analysis; (**I**) collagen analysis. (**B**–**G**) Analysis: * *p* < 0.05; ** *p* < 0.01; *** *p* < 0.001, **** *p* < 0.0001 one-way ANOVA vs. G2. (**H**) Analysis of E-cadherin levels; (**I**) Pim1 level; (**J**) α-SMA levels; (**K**) p-Smad3 levels. (**L**) Analysis of Snail protein levels. (**H**–**L**) Analysis: * *p* < 0.05, ** *p* < 0.01; *** *p* < 0.001; vs. G2, unpaired *t*-test.

ABI-171 effectively mitigated BLM-induced lung injuries, as indicated by improvements in Masson’s and H&E scores (Figure 2C–E). Notably, oral administration of ABI-171 (100 mg/kg) had similar efficacy compared to intranasal delivery (10 and 50 mg/kg doses), suggesting that both routes are viable, although the intranasal route seems to be more dose-effective. By day 21, mice treated with ABI-171 exhibited significant reductions in BLM-induced lung collagen and fibronectin levels (Figure 2F,G), underscoring the importance of targeting fibronectin in IPF management. Considering the similar results obtained between groups 4, 5, and 6, we further investigated the mechanism of action using only tissues from Group 4. PIM1 is upregulated consistently in the fibrotic lung tissues of IPF patients and in mice’s lungs induced by intratracheal BLM administration; treatment with ABI-171 significantly reduced the level of this enzyme (Figure 2I). E-cadherin, crucial for maintaining epithelial integrity, is often downregulated in pulmonary fibrosis, leading to EMT and subsequent fibrotic tissue remodeling. Our analysis revealed that oral treatment with ABI-171 significantly elevated E-cadherin levels (Figure 2H), suggesting its potential to halt pulmonary fibrosis progression by preserving epithelial integrity [25].

Figure 2J–L show significant reductions in p-Smad3, αSMA, and Snail protein levels, indicating a significant decrease in fibrotic activity. p-Smad3 is a key signaling molecule in the TGF-β pathway [26], which promotes fibrosis; thus, its reduction suggests less active TGF-β signaling. Reductions in αSMA, a marker of myofibroblasts [27]—the primary cells responsible for fibrosis—implies decreased myofibroblast presence or activity. Snail, a transcription factor involved in EMT [28], also shows lower levels, indicating inhibition of EMT and fewer epithelial cells converting into fibrotic mesenchymal cells. Collectively, these reductions demonstrate that the therapeutic intervention effectively mitigates the processes driving fibrosis, potentially ameliorating IPF progression.

## 3. Discussion

The results of this study highlight the significant therapeutic potential of ABI-171 in treating idiopathic pulmonary fibrosis (IPF), a disease for which there are currently no curative treatments. Although our results are based on a bleomycin-induced pulmonary fibrosis model in mice, it is important to acknowledge that both nintedanib and pirfenidone—the two FDA-approved antifibrotic drugs for IPF—were developed using similar models [29,30]. Ongoing drug development for IPF continue to rely on these models to target the pathways involved in disease pathogenesis [31,32]. In this context, the dual inhibition of DYRK1A and PIM1 kinases by ABI-171 provides a comprehensive approach to addressing the critical pathological processes of IPF, including inflammation, fibroblast activation EMT, and ECM remodeling.

ABI-171 significantly improved survival rates and reduced collagen accumulation in both preventive and therapeutic models of bleomycin-induced IPF. In the preventive model (Figure 1), where treatment began immediately after bleomycin administration, ABI-171 effectively mitigated early fibrotic changes and inflammation. This was evidenced by the significant reduction in lymphocyte and neutrophil counts in the BALF, suggesting a robust anti-inflammatory effect mediated by DYRK1A inhibition. DYRK1A plays a pivotal role in regulating inflammatory responses and fibroblast activation, both essential factors in fibrosis progression [17].

In the therapeutic model (Figure 2), where treatment began after fibrosis had been established, ABI-171 continued to demonstrate strong efficacy. The compound reduced lung collagen and fibronectin levels, both key markers of fibrosis, and improved histopathological scores. Notably, ABI-171 outperformed EGCG, a naturally occurring catechin derived from green tea, which is currently undergoing Phase 1 clinical trials for IPF [24]. While EGCG has shown promise due to its anti-inflammatory and antifibrotic properties, its clinical application is limited by poor bioavailability and potential safety concerns with long-term use [13,14]. Furthermore, EGCG is widely available as a dietary supplement and through green tea consumption, which complicates its positioning as a pharmaceutical-grade therapeutic. ABI-171, a fluoro-catechin derivative, addresses these limitations by offering enhanced bioavailability, selectivity, and potency, making it a more viable candidate for clinical use in IPF (Table 2).

Mechanistically, ABI-171 exerts its anti-fibrotic effects through the inhibition of both DYRK1A and PIM1 kinases and potentially including LOXL2 and TGF-β signaling as well. By inhibiting DYRK1A, ABI-171 modulates critical signaling pathways, including STAT3, reducing the transcription of pro-fibrotic genes and inhibiting fibroblast activation. PIM1 inhibition by ABI-171 disrupts the EMT process, a crucial event in fibrosis progression, by downregulating transcription factors like Snail and αSMA. The observed increase in E-cadherin levels, which are essential for maintaining epithelial integrity, suggests that ABI-171 helps preserve epithelial characteristics and prevents their transition into a fibrogenic state.

Whether direct inhibition of LOXL2 by ABI-171, as well as inhibition of TGF-β signaling by the metabolite of ABI-171, resulted from interacting with LOXL2 has not yet been demonstrated; however, the structural characteristics of the compound raise the hypothesis that ABI-171 could affect LOXL2 and TGF-β signaling similarly as described for polyphenolic compounds [9]. LOXL2 is a key enzyme involved in collagen cross-linking and tissue stiffening, both hallmarks of fibrosis [33]. Inhibition of LOXL2 has been associated with decreased fibrotic tissue formation in various models, and its regulation is tied to the TGF-β signaling pathway [10]. Given that ABI-171 significantly reduces levels of Smad3—a critical downstream effector of TGF-β signaling—and other transcription factors involved in EMT [34], it is plausible that ABI-171 may also reduce LOXL2-mediated collagen deposition and ECM remodeling. This hypothesis is further supported by ABI-171’s ability to reduce Snail expression, which is known to be regulated by LOXL2, as well as TGF-β, during EMT [34]. However, further studies are required to confirm any direct or indirect inhibition of LOXL2 and TGF-β signaling by ABI-171.

An intriguing observation in this study was the differential impact of intranasal administration on body weight in the preventive versus therapeutic models. In the preventive model, intranasal ABI-171 significantly improved body weight, likely due to its potent anti-inflammatory effects during the early stages of fibrosis. However, in the therapeutic model, where fibrosis had already been established, the primary benefit of ABI-171 was a reduction in fibrosis rather than body weight restoration. This variance highlights the importance of early intervention in IPF and underscores the distinct pathological processes at play in the early versus late stages of the disease.

ABI-171’s superior pharmacokinetic profile compared to EGCG and ABI-154, characterized by improved exposure and lung tissue penetration, also plays a crucial role in its efficacy (Table 2). Intranasal administration ensures high local concentrations of the drug in the lungs, the primary site of action, while minimizing systemic exposure and potential side effects. Utilizing intranasal delivery as a surrogate for intratracheal delivery or direct delivery to the lung (i.e., inhalation), this approach effectively bypasses challenges associated with first-pass metabolism and food-related interactions, common limitations of oral delivery systems, particularly for polyphenols like EGCG. As reported by Epstein-Shochet et al. [35], intratracheal administration has been associated with side effects such as tracheal injury and challenges in animal recovery, highlighting the benefits of intranasal delivery as a safer alternative.

In addition to ABI-171, we also evaluated ABI-154, another catechin derivative. While ABI-154 demonstrated some efficacy in initial preclinical tests by modulating inflammatory and fibrotic pathways [15], its poorer pharmacokinetic properties and limited exposure [16] restricted its therapeutic impact (Table 2). These limitations highlight the enhanced potential of ABI-171, which was developed to overcome the issues seen with ABI-the cor154.

In conclusion, ABI-171 represents a promising disease-modifying therapy for IPF by addressing multiple pathological processes, including inflammation, fibroblast activation, EMT, and ECM remodeling through the dual inhibition of DYRK1A and PIM1 kinases, as well as LOXL2 and TGF-β signaling inhibition. Its superior pharmacokinetic properties and multi-targeted mechanism of action make ABI-171 a transformative therapeutic agent. Further clinical trials are warranted to validate these preclinical findings and fully explore the therapeutic potential of ABI-171 in patients with IPF. The success of ABI-171 in these preclinical models provides a strong foundation for its advancement into clinical development, with the potential to significantly improve outcomes for patients suffering from this devastating disease.

## 4. Materials and Methods

### 4.1. Materials and Reagents

Bleomycin (Selleck Chemicals, Houston, TX, USA, Cat: S1214) was used as the fibrosis-inducing agent. CMC-Na (carboxymethyl cellulose sodium) was sourced from Sigma-Aldrich (St. Louis, MO, USA, Cat: C4888-500G) as a vehicle solution. Pirfenidone was obtained from Bide Pharmatech (Shanghai, China, Cat: BD28958-5g) for positive control treatment. (2-Hydroxypropyl)-β-cyclodextrin (HP-β-CD) was supplied by MedChemExpress (Monmouth Junction, NJ, USA, Cat: HY-101103), and polyethylene glycol 400 (PEG400) from Sigma-Aldrich (Cat: 81172) was used as a solvent. Na_2_EDTA (disodium ethylenediaminetetraacetic acid) from Sigma-Aldrich (Cat: E5134-50G) was used in reagent preparations. ABI-154 and ABI-171 (purity 98%) were synthesized as described in our previous publication, denoted as compounds **1b** and **1c**, respectively [16]. The solutions for drug administration were prepared in PEG400 and HP-β-CD mixtures as described in [15,16]. Hematoxylin for histological analysis was purchased from ZSGB-BIO (Beijing, China, Cat: ZLI-9610).

### 4.2. ABI-171 Pharmacokinetics and Lung-Tissue Distribution

Female C57BL/6 mice with body weights of 17–24 g were purchased and maintained in air-conditioned quarters with 12 h light/dark cycles. They were given a commercial mice chow and water ad libitum. The experiments started after acclimation for at least 1 week. Dosing solutions were made fresh. Fasted mice were administered the test drug by oral (PO), intranasal (IN), and intravenous (IV) routes. The vehicle for the IN and PO routes was composed of 12% PEG400, 10% HP-β-CD, and saline q.b.; for IV, water was used. The compounds were delivered using a single IV dose of 50 μL (5 mg/kg), an IN dose (60 mg/kg, 5 μL solution/nostril/twice), and a single PO dose of 10 mL/kg (100 mg/kg), as previously described [16]. Post-dosing, ~500 µL of blood samples were collected (cardiac puncture) from three mice at each time point at 0.5, 1, 2, 4, 6, 8, and 24 h under isoflurane anesthesia. Blood was collected into K_2_EDTA microtubes, kept on ice all the time and centrifuged within 30 min of collection. Blood samples in EDTA vacuum tubes were centrifuged at 4 °C at 15,000× *g* for 4 min; ~250 µL of plasma was collected into polyethylene tubes containing 50 µL of the ascorbic acid (AA)/TCEP stabilizing solution (20 mM AA and 13 mM TCEP in 50 mM K_2_HPO_4_ buffer. The pH of the solution was adjusted to 6.5 using 2 M NaOH). After collection, the plasma samples were immediately snap-frozen in isopropanol/carbon-dioxide dry ice and stored at −80 °C until analysis.

Test catechin was extracted from the above-collected plasma through 3 successive additions of 350 µL of acetonitrile. The plasma and solvent mixture were vortex-mixed for 2 min then snap-frozen with isopropanol/carbon-dioxide dry ice and the extraction solvent was removed. The solvent fractions were pooled into a polyethylene tube kept on ice. The combined fractions were evaporated under a gentle stream of nitrogen at ambient temperature. The residue obtained after evaporation was reconstituted in 200 µL of 75 mM citric acid/25 mM ammonium acetate: acetonitrile (75:25, by vol), vortexed vigorously for 5 min, and 20 µL of the resulting solution was injected into the HPLC column. Lung tissue was collected and treated immediately to avoid product degradation. Within 5 min of extraction, about ~0.4 g of the tissue was homogenized with 1 mL of ice-cold 0.4 M sodium phosphate buffer containing 6 mg of ascorbic acid and 0.5 mg of Na_2_EDTA (final pH of 6.5). After centrifugation at 4 °C at 15,000× *g* for 4 min, the supernatant was collected into polyethylene tubes containing 50 µL of the AA/TCEP stabilizing solution (20 mM AA and 13 mM TCEP in 50 mM K_2_HPO_4_ buffer. The pH of the solution was adjusted to 6.5 using 2 M NaOH), immediately snap-frozen in isopropanol/carbon-dioxide dry ice and stored at −80 °C until analysis. Extraction was performed as described above for plasma.

### 4.3. Efficacy Studies: Bleomycin-Induced Animal Models of IPF

To evaluate the therapeutic potential of ABI-154 and ABI-171, we utilized two well-established bleomycin-induced animal models of IPF: a preventive model and a therapeutic model. These models are widely recognized for their ability to mimic the fibrotic processes observed in human IPF, including inflammation, fibroblast proliferation, and excessive deposition of ECM proteins.

#### 4.3.1. Preventive Model

In the preventive model, male C57BL/6 mice, aged 8–10 weeks, were used to simulate the early intervention scenario where treatment begins immediately following the induction of lung injury. The animals were randomly assigned to different groups (*n* = 6 per group): Control (normal), BLM (vehicle), pirfenidone (100 mg/kg orally, thrice daily), ABI-154 (60 mg/kg intranasally, once daily), and ABI-171 (60 mg/kg intranasally, once daily). On day 0, pulmonary fibrosis was induced by administering a single intratracheal infusion of bleomycin at a dose of 3 U/kg body weight, dissolved in 50 μL of sterile saline. Treatment with the test compounds commenced immediately after bleomycin administration and continued daily for 21 days. Body weight, survival rate, and clinical signs were monitored throughout the study.

At the end of the study period, the mice were sacrificed, and their lungs were harvested for histological and biochemical analyses. Lung tissues were fixed in 10% formalin and embedded in paraffin for histological examination using hematoxylin and eosin (H&E) staining to assess tissue morphology, and Masson’s trichrome staining to evaluate collagen deposition. Additionally, lung hydroxyproline content, a marker of collagen accumulation, was quantified using a colorimetric assay. Inflammatory cell infiltration was assessed by counting lymphocytes and neutrophils in bronchoalveolar lavage fluid (BALF).

#### 4.3.2. Therapeutic Model

In the therapeutic model, the efficacy of ABI-171 was assessed in a delayed treatment scenario in which the intervention began after the establishment of lung fibrosis. Male C57BL/6 mice, aged 8–10 weeks, were randomly assigned to the following groups (*n* = 6 per group): Control (normal), BLM (vehicle), EGCG (100 mg/kg orally, once daily), and ABI-171 (intranasally at 10 mg/kg and 50 mg/kg once daily, or orally at 100 mg/kg once daily). Pulmonary fibrosis was induced by a single intratracheal infusion of bleomycin at a dose of 3 U/kg body weight. Seven days post-bleomycin administration, when fibrosis was established, treatment with the test compounds commenced and continued daily for an additional 21 days.

Throughout the treatment period, body weight, survival rate, and clinical signs were monitored. At the study’s conclusion, mice were sacrificed, and their lungs were harvested for comprehensive histological and biochemical analyses. Lung tissues were processed for H&E and Masson’s trichrome staining to evaluate fibrosis and collagen deposition. Lung hydroxyproline content was measured to quantify collagen accumulation. Inflammatory cell infiltration in BALF was assessed by counting lymphocytes and neutrophils. Additionally, the expression levels of fibrosis-related markers, including PIM1, E-cadherin, p-Smad3, α-SMA, and Snail, were analyzed using Western blotting and immunohistochemistry to elucidate the molecular mechanisms underlying the anti-fibrotic effects of ABI-171.

These two complementary bleomycin-induced models allowed us to assess the preventive and therapeutic efficacy of ABI-154 and ABI-171, providing a robust framework for evaluating their potential as treatments for IPF.

### 4.4. Statistical Analysis

Results are expressed as mean ± SD. All data were analyzed using GraphPad Prism Version 10.0 software. Statistical comparisons were performed using Student *t*-test for two-group comparisons and one or two-way ANOVA with a Tukey’s post hoc test for multiple comparisons. *p* < 0.05 was considered to be statistically significant.

## Figures and Tables

**Table 1:** Best DYRK1A inhibitors.

Code	Structure	DYRK1A IC_50_	PIM1 IC_50_	PIM2 IC_50_	PIM3 IC_50_
EGCG	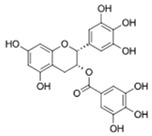	235 nM	1359 nM	N/A	N/A
ABI-171	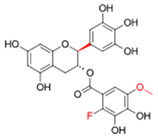	73 nM	87 nM	255 nM	813 nM
ABI-154	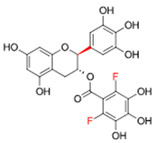	28 nM	722 nM	1081 nM	6000 nM

**Table 2:** Best fluoro catechins pharmacokinetic parameters. NRV: not reportable value; Cmax = highest plasma concentration; AUC = Area under-the-curve; CL = total plasma clearance; CL/F = apparent total clearance after IN or PO administration; Vz = apparent volume of distribution of terminal phase after bolus administration; Vz/F = apparent volume of distribution of terminal phase after IN or PO administration; F% = absolute oral bioavailability (PO/IV).

		ABI-171					ABI-154	EGCG	
Parameter	Unit	IV (5 mg/kg)	IN (60 mg/kg)		PO (100 mg/kg)		PO (100 mg/kg)	IV (10 mg/kg)	PO (100 mg/kg)
		Plasma	Plasma	Lung	Plasma	Lung	Plasma	Plasma	Lung
T_1/2_	h	3.2	4.8	3.93	4.7	NRV	12.7	5.7	5
T_max_	h		2	4.00	1	1.00	0.5		
T_last_	h		24	24	24	24	24		
C_max_	ng/mL (or ng/g)	657	2353	8739	684	231	131	4417	55
AUC_0-t_	ng h/mL	1276	15,763	23,067	4174	214	379	1798	360
AUC_(0-inf)_	ng h/mL	1393	16,079	23,210	4275	NRV	472	1826	395
Vz(IV) or Vz/F ((IN and PO)	L/kg	17			15.6		NA	45.2	2680
CL(IV) or CL/F (IN and PO)	mL/min/kg	60			101		NA	5.5	253
F	%				16		NA		2

## Data Availability

Data is contained within the article.
